# Seasonal Variation and Possible Biosynthetic Pathway of Ginsenosides in Korean Ginseng *Panax ginseng* Meyer

**DOI:** 10.3390/molecules23071824

**Published:** 2018-07-23

**Authors:** Dongmin Kim, Mihyang Kim, Gem Stephen Raña, Jaehong Han

**Affiliations:** Metalloenzyme Research Group and Department of Plant Biotechnology, Chung-Ang University, Anseong 17546, Korea; casualpe@gmail.com (D.K.); mihcoterie@gmail.com (M.K.); gemstephenrana@gmail.com (G.S.R.)

**Keywords:** *Panax ginseng*, ginsenosides, UHPLC, seasonal variation, biosynthesis

## Abstract

Whereas Korean ginseng, *Panax ginseng* Meyer, is harvested in the fall, the variation of ginsenoside content in field-grown ginseng across seasonal development has never been investigated in Korea. Thus, ultra-high performance liquid chromatography (UHPLC) analysis of nine major ginsenosides, including ginsenoside Rg1, Re, Rf, Rg2, Rb1, Rc, Rb2, Rd, and Ro, in the roots of five-year-old *P. ginseng* cultivated in Bongwha, Korea in 2017 was performed. The total ginsenoside content changed as many as three times throughout the year, ranging from 1.37 ± 0.02 (dry wt %) in January to 4.26 ± 0.03% in May. Total ginsenoside content in the harvest season was 2.49 ± 0.03%. Seasonal variations of panaxadiol-type ginsenosides (PPD) and panaxatriol-type ginsenosides (PPT) were found to be similar, but more PPD was always measured. However, the seasonal variation of oleanolic acid-type ginsenoside, Ro, was different from that of PPD and PPT, and the highest Ro content was observed in May. The ratio of PPD/PPT, as well as other representative ginsenosides, was compared throughout the year. Moreover, the percent composition of certain ginsenosides in both PPD and PPT types was found to be in a complementary relationship each other, which possibly reflected the biosynthetic pathway of the related ginsenosides. This finding would not only provide scientific support for the production and quality control of the value-added ginseng products, but also facilitate the elucidation of the ginsenoside biosynthetic pathway.

## 1. Introduction

Korean ginseng (*Panax ginseng* Meyer), as one of the most profitable medicinal plants in the agricultural industry, is usually harvested in October after a four- to six-year cultivation. Traditionally, ginseng has been used for tonic, sedative, anti-fatigue, and anti-gastric ulcer drugs. The anti-diabetic and anticancer activities of ginseng have also drawn increasing attention recently [1]. Ginseng is also considered as a foodstuff, and a plethora of health products and general foods involving ginseng are produced in Korea under the governmental regulations. In 2016, 20,386 tons of fresh ginseng were harvested, worth 700 million dollars [2]. However, the quality of fresh ginseng is determined not by the content of ginsenosides but by the appearance and size when fresh ginseng is marketed. The dried and heat-processed ginseng roots, Radix Ginseng and Radix Rubra Ginseng, respectively, are listed on the pharmacopeias of many countries, including Korea [3], Japan [4], and China [5]. Ginsenosides are believed to be the major bioactive secondary metabolites of ginseng, responsible for the various beneficial health promoting effects. The marker compounds of ginseng and red ginseng monitored by the Korean pharmacopoeia include ginsenoside Rb1, Rg1, Re, and Rg3 [3]. The European pharmacopoeia requires the determination of eight ginsenosides of ginsenoside Rg1, Re, Rf, Rg2, Rb1, Rc, Rb2, and Rd [6].

Analysis of ginsenosides with different cultivation years has been a major issue in ginseng research to verify whether the cultivation year is related to the content of ginsenosides. For example, the total ginsenosides of *P. ginseng* cultivated in Jilin, China, including ginsenoside Rg1, Re, Rb1, Rc, Rb2, Rb3, and Rd, were reported to increase from the first year to the fifth year [7]. On the other hand, the content of ginsenosides appears to be influenced more by cultivation region than by cultivation year when compared between four-year and five-year ginsengs [8]. Therefore, it is still inconclusive whether the cultivation year of ginseng is a key factor determining the quality of ginseng products. 

In the meantime, the variation of ginsenoside content of ginseng across seasons has been studied by a few research groups. Wu et al. determined the ginsenoside contents of three-year-old American ginseng (*P. quinquefolius*) roots, and reported the highest amount of total ginsenoside at the leaf-development stage [9]. Kim et al. has analyzed the ginsenoside changes of hydroponically grown *P. ginseng* during foliation and reported the highest total ginsenoside content from the intermediate leaf stage [10]. However, the number of measurements is small and the samples used for these studies do not represent field-grown ginseng in the literature, so it is difficult to generalize the results to field-cultivated ginseng. The most relevant study was reported in 2013 by Chen et al. [11]. Five-year-old *P. ginseng* cultivated in Jilin, China was analyzed for four months, between May and September. The authors showed that the content of ginsenoside changed significantly and the total ginsenoside content decreased during the growth. However, the cultivation practices and climate are very different between China and Korea.

The study of the variation of ginsenoside content during the annual growth can help elucidate the relationship between the growth stage and the quality of ginseng. In addition, it can improve the practice of ginseng cultivation through developing value-added ginseng products. Furthermore, the detailed compositional changes of ginsenosides can encourage research relevant to the ginsenoside biosynthetic pathway, which is relatively little studied. To the best of our knowledge, there has been no extensive study on the seasonal variation of ginsenosides with cultivated Korean ginseng. Therefore, the seasonal variation of nine major ginsenosides (Table 1) in the five-year-old *P. ginseng* cultivated in Bongwha, Korea was investigated.

## 2. Results

### 2.1. Analytes Preparation and UHPLC Analysis of P. ginseng

**Figure 1:**
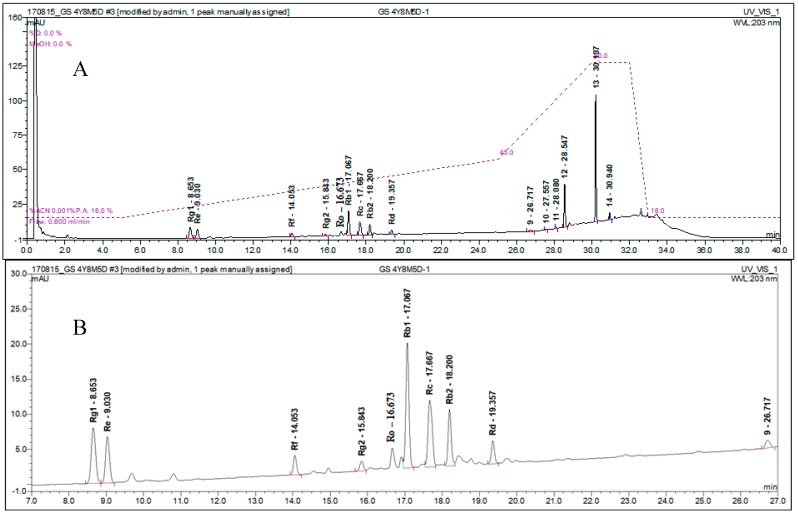
UHPLC chromatogram (203 nm) of the ginseng analytes (**A**) and the expansion (**B**) showing nine ginsenosides.

The analysis of ginsenosides, especially from fresh ginseng, is known to be influenced by many experimental conditions, such as the sample and analyte preparation processes [12,13]. Based on the existing pharmacopeias and literature, we have developed a simple and reproducible experimental procedure for the chromatographic analysis of nine ginsenosides. Aqueous methanol (80%), without any other reagents, was used for the extraction solvent at 40 °C, and the analyte preparation time was minimized to prevent systematic errors. With repetitive trials, the amount of dried ginseng powder sample was reduced to 50 mg, which guaranteed reproducible analysis of ginsenosides. Ginsenosides Rg1, Re, Rf, Rg2, Rb1, Rc, Rb2, Rd, and Ro, representing panaxatriol-type ginsenosides (PPT), panaxadiol-type ginsenosides (PPD), and oleanolic acid-type ginsenosides, were selected for the UHPLC analysis of ginsenosides seasonal changes. These nine ginsenosides were the most abundant ginsenosides in the five-year ginseng roots. Figure 1 gives a typical UHPLC chromatogram of a fresh ginseng sample resolved into nine ginsenosides, which were observed at retention times between 8 and 20 min. The reproducible quantitative UHPLC analysis was obtained by using the calibration curves of the standard ginsenosides shown in Table 1.

### 2.2. Seasonal Changes of Ginsenosides

**Figure 2:**
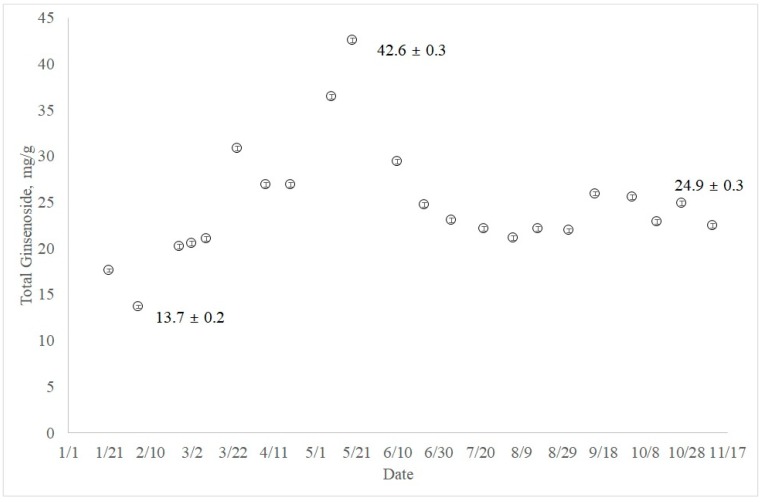
Total ginsenoside changes of five-year-old ginseng throughout the year.

Beginning in January 2017, the total ginsenoside contents were measured throughout the year. To our surprise, the highest content of nine total ginsenosides was observed at the end of May (Figure 2). The high ginsenoside content (42.6 ± 0.3 mg/g) in May decreased until August, and steadily increased in the fall (24.9 ± 0.3 mg/g). Interestingly, the total ginsenoside content was also high at the end of March, with a value of 30.9 ± 0.2 mg/g.

When the nine ginsenosides in each ginseng sample were compared, it was found that the relative distributions were generally consistent throughout the year (Table 2). For example, ginsenoside Rb1 was the most abundant ginsenoside throughout the year, followed by ginsenoside Rc. The presence of ginsenoside Rf has been considered a characteristic trait of Korean ginseng. The average content of ginsenoside Rf was less than 1 mg/g, but highest on May 19 with an amount of 1.30 ± 0.06 mg/g. 

**Figure 3:**
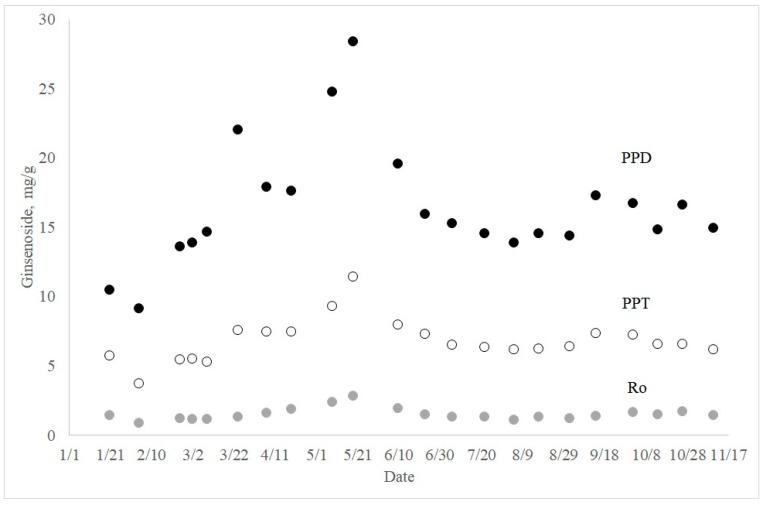
PPD, PPT, and ginsenoside Ro changes of five-year-old ginseng throughout the year.

Among PPD, the concentration of each ginsenoside always followed the order Rb1 > Rc > Rb2 > Rd. The relative distribution of PPT was found to be Rg1 ≈ Re > Rf > Rg2 for most samples. However, the order of abundance was rather inconsistent for PPT. Seasonal changes of PPT, PPD, and Ro showed similar patterns. Both PPD and PPT contents were high in May, with values of 28.4 ± 0.2 mg/g and 11.4 ± 0.1 mg/g, respectively. PPD was also high in March (22.0 ± 0.2) (Figure 3). The highest content of ginsenoside Ro was also observed in the middle of May, with a measured value of 2.83 ± 0.06 mg/g.

### 2.3. Seasonal Variation of Ginsenoside Composition

**Figure 4:**
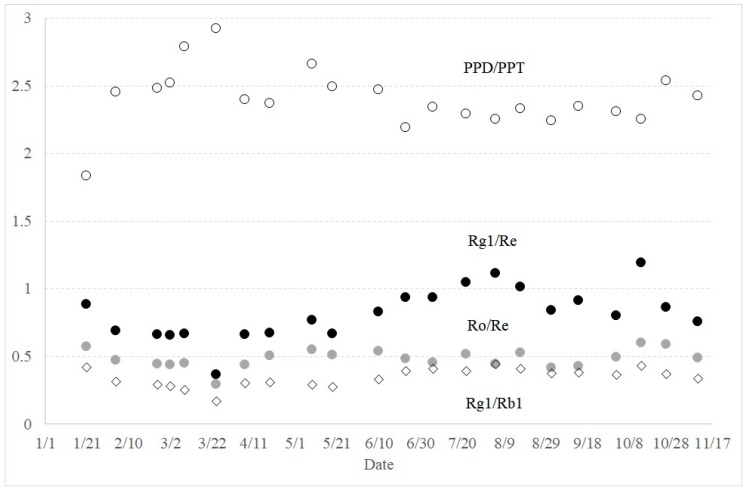
Seasonal variations of the ratios of PPD over PPT (open circle), ginsenosides Rg1 over Re (closed circle), ginsenosides Ro over Re (shaded circle), and ginsenoside Rg1 over Rb1 (diamond) throughout the year.

There have been numerous efforts to correlate the ginsenoside composition to the characteristics of ginseng, such as biological activity. The ratios of the specific ginsenosides were calculated from the data shown in Table 2. The ratio of PPD/PPT was considered a key parameter determining the different biological activity of ginseng [14]. The ratio of PPD/PPT throughout the year was found to be about 2.5. It was highest at the end of March and decreased until October (Figure 4). The ratio of Rg1 and Rb1 was also proposed as a criterion to specify the ginseng species [8]. The observed ratio of Rg1/Rb1 was about 0.3 throughout the year. Rg1 and Rb1 were also suggested as antagonists with regard to angiogenesis [15].

Recently, the ratio of Ro and Re was suggested as a characteristic marker of ginseng ages and the ratio of Rg1 and Re was proposed as a characteristic marker of the harvest season [16]. Throughout the year, the ratio of Ro over Re was found to be around 0.5. It increased in October, but was distinctly low in March (Figure 4). The ratio of Rg1 over Re appears to increase during the vegetation period from May to August, but was highest in the middle of October with a ratio of 1.2. However, the ratio changes were distinctively different from the reported behaviors [16].

**Figure 5:**
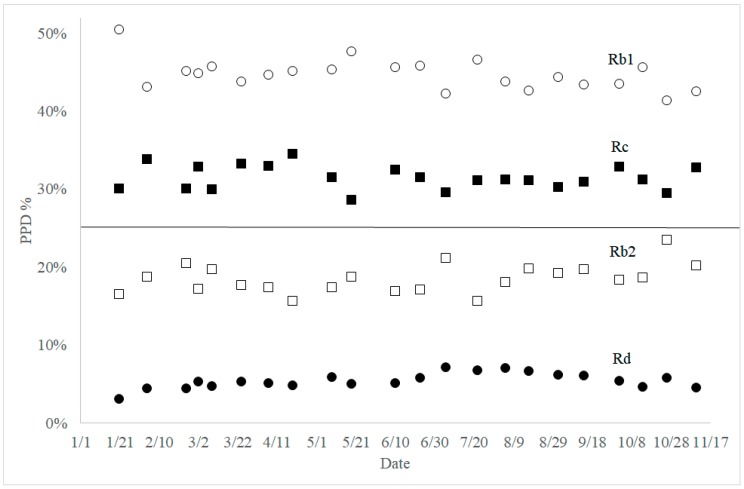
Seasonal composition changes of PPD ginsenosides Rb1 (open circle), Rc (closed square), Rb2 (open square), and Rd (closed circle). PPD percentage was calculated from the sum of four PPD ginsenosides.

**Figure 6:**
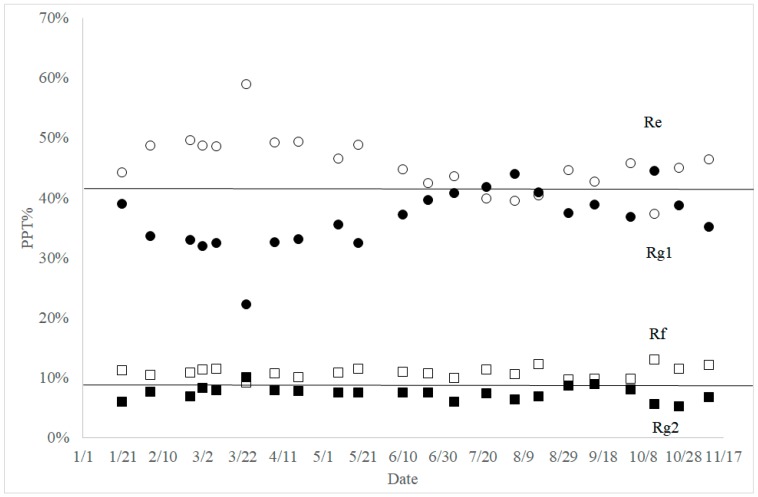
Seasonal composition changes of PPT ginsenosides Re (open circle), Rg1 (closed circle), Rf (open square), and Rg2 (closed square). PPD percentage was calculated from the sum of four PPD ginsenosides.

When the percent composition of ginsenosides was compared in each group of ginsenosides, interesting results were obtained. In the PPD group, the percentages of ginsenoside Rb1+Rd and Rc+Rb2 were always close to 50%. Moreover, percentage changes between ginsenoside Rb1 and Rd, and also between ginsenoside Rc and Rb2, were almost mirror images (Figure 5). In the PPT ginsenosides, the percentage of ginsenoside Rg1 + Re was always between 80% and 84%. The change patterns of ginsenoside Rg1 and Re percentage over the year were almost identical (Figure 6). A similar trend was also observed between those of ginsenosides Rf and Rg2.

## 3. Discussion

Korean ginseng, *P. ginseng* Meyer, has been used as a traditional medicine in Asia for a long time. Nowadays, the plants in the genus *Panax* are cultivated widely around the world as an important agricultural crop. With the increased industrial applications and the world commercial market, scientific research related to ginsenoside analysis has been developed. Because most ginseng products are produced from four- to six-year-old ginseng roots, the quantitative analysis of ginsenosides in fresh ginseng has been focused on the ages of ginseng to assist with the development of value-added ginseng products through physical, chemical, and biological processes [17,18]. 

In this study, analysis of nine ginsenosides has been performed with five-year-old ginseng cultivated in Bongwha, Korea, throughout the year, because a seasonal change in ginsenosides has never been reported in cultivated Korean ginseng. Although the ginsenoside content increase over the cultivation years is still disputable [7,8], the content of ginsenosides seems to change consistently during the growth. From the literature, total ginsenosides of three-year-old American ginseng were highest (17.05 mg/g) at the leaf-stem separation stage [9]. It was also reported that the total ginsenosides of fine roots were highest (20.06 ± 0.70 mg/g) at the intermediate leaf stage from the hydroponically grown three-year-old Korean ginseng [10]. From our study, the highest total ginsenoside content (42.57 ± 0.26 mg/g) was observed at the end of May (Figure 2). It is not sure whether the ginseng growth stage can be related to the specific time, but the month of May is the best harvest season for Korean ginseng when only the total ginsenoside content is considered. 

Overall, the relative composition of nine ginsenosides did not change significantly through the year. However, each group of ginsenosides, such as PPD, PPT, and oleanolic acid-type ginsenoside Ro, exhibited distinctively different seasonal variations. For example, all three types of ginsenoside increased at the end of March, but the extent of increase was most significant in PPD. During April, the amounts of PPD and PPT decreased, but the amount of ginsenoside Ro slightly increased. These changes in the relative composition of ginsenosides are believed to represent the biosynthetic pathway of ginsenosides. Although the biosynthetic pathway of ginsenosides has been little studied, a rational pathway for the studied ginsenosides can be suggested based on the molecular structures. 

The ratios of Rg1/Re and PPD/PPT have been considered key parameters of ginseng quality control and biological activity, respectively. For example, it was proposed that PPT induces cyclic GMP and NO production [19]. Hence, there were efforts to explain the “hot feeling” of ginseng consumption based on the ratio of PPD over PPT, because those signal molecules result in the relaxation of the aorta [14]. Similarly, the low PPD/PPT ratio, characteristic of red ginseng, was proposed to enhance the spatial working memory [20]. From our study, the ratio of PPD over PPT was always more than 2, except in January, when the ratio was 1.8. The ratio of PPD/PPT was highest at the end of March, with a value of 2.9. Another study reported very different PPD/PPT ratios from different ages of *P. ginseng*, close to 1.0 [21]. The changes of ginsenoside Rg1 and Rb1 were proposed to be related to the ages of ginseng [10]; however, such trends were not observed from our study. It appears that the content of each ginsenoside was influenced more by the growth stage than by the age of the ginseng.

**Figure 7:**
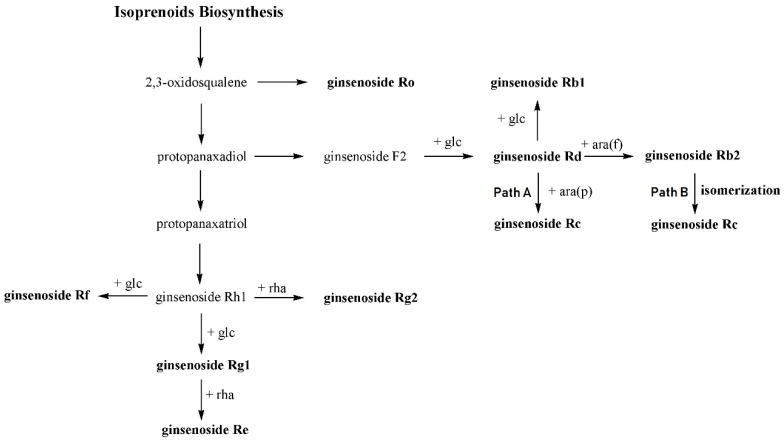
Proposed biosynthetic pathway for nine ginsenosides. Instead of arabinopyranosyl transfer, isomerization of Rb2 is proposed for the biosynthesis of ginsenoside Rc.

From the analysis of nine ginsenosides throughout the year, interesting points were observed among the biosynthetically related group. In the PPD ginsenosides, the percentages of ginsenoside Rb1 + Rd and that of ginsenoside Rc + Rb2 were always close to 50% (Figure 5). Moreover, a complementary relationship in the percent compositions was observed between ginsenosides Rb1 and Rd, also between ginsenosides Rc and Rb2. Based on the molecular structures of ginsenosides and glycosylation steps, a reasonable biosynthetic pathway for the ginsenosides can be proposed (Figure 7). As shown in Figure 7, sequential glucose transfer to ginsenoside F2 would result in ginsenosides Rd and Rb1. Arabinofuranosyl transfer to ginsenoside Rd would also lead to the formation of ginsenoside Rb2. Theoretically, two pathways for the biosynthesis of ginsenoside Rc are possible, from ginsenoside Rd (path A) and Rb2 (path B).

**Figure 8:**
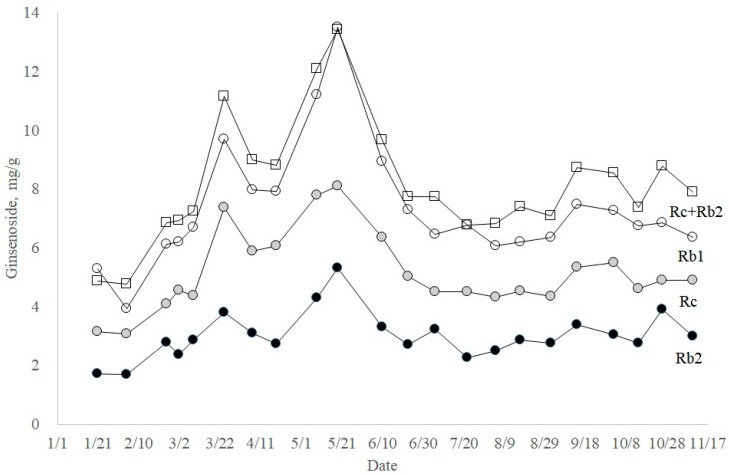
Seasonal changes of PPD ginsenosides Rc and Rb2 (open square), Rb1 (open circle), Rc (shaded circle), and Rb2 (closed circle).

It appears that the biosynthesis of ginsenosides Rc and Rb2 uses the same pool of biosynthetic precursors because of the complementary relationship shown in Figure 5. One of the possible explanations for this correlation would be that ginsenosides Rc and Rb2 use the same biosynthetic precursor, ginsenoside Rd. From Figure 8, it is found that the sum of ginsenosides Rc and Rb2 changed analogously to Rb1, even though ginsenoside Rc was more abundant than ginsenoside Rb2 throughout the year. Therefore, it is proposed that ginsenoside Rc is biosynthesized from ginsenoside Rd using path A in Figure 7. It was recently reported that UDP-arabinose mutase catalyzes the formation of UDP-arabinosylfuranose from UDP-arabinosylpyranose, which may be common in plants [22]. 

In the PPT ginsenosides, the percentage of ginsenoside Rg1 + Re was always between 80% and 84%, and the changes of ginsenoside Rg1 and Re percentages through the year were almost symmetric (Figure 6). It appears that ginsenoside Rg1 is the biosynthetic precursor of ginsenoside Re biosynthesis, because the complementary relationship between the two ginsenosides remained constant regardless of flux changes. A similar conclusion could be drawn for ginsenosides Rf and Rg2, which supports the PPT biosynthetic pathway in Figure 7.

## 4. Materials and Methods 

### 4.1. Materials

The ginsenoside mixture of Rg1, Re, Rf, Rg2, Rb1, Rc, Rb2, and Rd (0.1 g/L) was purchased from Cerilliant Co. (Round Rock, TX, USA), and ginsenoside Ro were purchased from Embo Laboratory (Daejeon, Korea). Voucher specimens of ginseng samples were deposited to Phytobean AC LTD (Yecheon-gun, Korea). HPLC-grade methanol (MeOH), water, and acetonitrile (MeCN) were purchased from Honeywell (Morris Plains, NJ, USA). Analytical grade phosphoric acid was purchased from Sigma-Aldrich (St. Louis, MO, USA).

### 4.2. Ginseng Samples and Analyte Preparation

The *P. ginseng* samples were collected from the ginseng field at Bongwha, Korea (36°85′2″ N; 128°80′63″ E; altitude 280 m) in 2017. The number of ginseng samples was at least 20. On the day of harvest, the root parts were cleaned with water to remove the soil and sliced. The samples were dried under shade at room temperature until no changes in weight were observed. The dried ginseng root samples were powdered for the analysis. 

For the analysis of ginsenosides by UHPLC, 50.0 mg of meshed dried ginseng powder was added to 10.0 mL of 80% methanol and the solution was sonicated with 135 kHz for 2 h at 40 °C. Then, the solution was centrifuged for 15 min (2000× *g*), and 8 mL of supernatant were taken for the dryness under vacuum with centrifugation (3200× *g*, 120 min, 60 °C). The residue was dissolved in 2.0 mL of MeOH and filtrated through a 0.22-μm cellulose syringe filter (Grace Co., Columbia, MD, USA) for UHPLC analysis. 

### 4.3. General UHPLC Method

A Thermo UHPLC 3000 with a PDA detector, equipped with a C18 Phenomenex Kinetex™ (Torrance, CA, USA) column (2.1 × 100 nm, 1.7 μm), was used for UHPLC analysis. The injection volume was 2 μL and the flow rate was 0.6 mL/min. The mobile phase for the analysis of ginsenosides was composed of 0.001% phosphoric acid in deionized water (solvent A) and 0.001% phosphoric acid in MeCN (solvent B). For the eluent gradient system, solution B started at 18% for 5 min and increased linearly to 45% from 5 min to 25 min, to 90% from 25 min to 30 min, and was held constant at 90% for 2 min before returning to the initial composition. The column was maintained at 40 °C. 

### 4.4. Calibration Curves of Ginsenosides

The standard solutions of ginsenosides were diluted to five to nine different concentrations between 2 and 100 μg/mL, depending on the abundance in the ginseng sample. The analytes were then analyzed by UHPLC in triplicate, and the calibration curves were constructed by plotting peak area versus the concentration of ginsenoside (Table 1).

## 5. Conclusions

Variation of ginsenoside content in field-grown Korean ginseng across seasonal development has been analyzed by UHPLC for the first time in Korea. The total ginsenoside content, sum of nine major ginsenosides, changed as many as three times throughout the year, ranging from 1.37 ± 0.02 (dry wt %) in January to 4.26 ± 0.03% in May. Total ginsenoside content in the harvest season was only 2.49 ± 0.03%. The results will not only provide scientific support for the production and quality control of the value-added ginseng products, but also facilitate the elucidation of the ginsenoside biosynthetic pathway.

## Figures and Tables

**Table 1:**
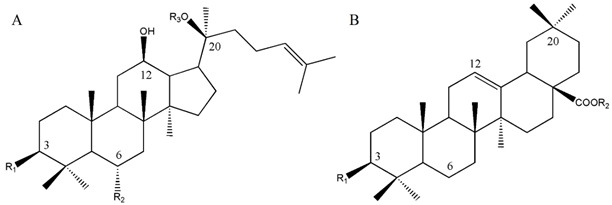
Structure and calibration curves of nine ginsenosides studied in this study.

Ginsenoside	Structure	R1	R2	R3	Calibration Curve ^1^	RC ^2^	LOD (mg/g) ^3^	LOQ (mg/g) ^4^
Rb1	A	–*O*–Glc^2^–^1^Glc	–H	–Glc^6^–^1^Glc	*y*(±0.01006) = (0.02027 ± 0.00007)*x* + (−0.00621 ± 0.00282)	0.99975	0.092	0.305
Rb2	A	–*O*–Glc^2^–^1^Glc	–H	–Glc^6^–^1^Ara(p)	*y*(±0.00974) = (0.01742 ± 0.00006)*x* + (−0.00851 ± 0.00273)	0.99974	0.098	0.324
Rc	A	–*O*–Glc^2^–^1^Glc	–H	–Glc^6^–^1^Ara(f)	*y*(±0.01099) = (0.02164 ± 0.00007)*x* + (−0.00499 ± 0.00308)	0.99978	0.099	0.328
Rd	A	–*O*–Glc^2^–^1^Glc	–H	–Glc	*y*(±0.01105) = (0.02182 ± 0.00007)*x* + (−0.00557 ± 0.00310)	0.99977	0.114	0.380
Rg1	A	–OH	–*O*–Glc	–Glc	*y*(±0.00872) = (0.01429 ± 0.00005)*x* + (−0.00029 ± 0.00241)	0.99967	0.107	0.356
Re	A	–OH	–*O*–Glc^2^–^1^Rha	–Glc	*y*(±0.00711) = (0.01468 ± 0.00005)*x* + (−0.00526 ± 0.00199)	0.99981	0.108	0.361
Rf	A	–OH	–*O*–Glc^2^–^1^Glc	–H	*y*(±0.00794) = (0.01560 ± 0.00005)*x* + (−0.00210 ± 0.00223)	0.99978	0.095	0.318
Rg2	A	–OH	–*O*–Glc^2^–^1^Rha	–H	*y*(±0.01072) = (0.01769 ± 0.00007)*x* + (−0.00682 ± 0.00301)	0.99968	0.090	0.298
Ro	B	–*O*–Glc^2^–^1^Glc	–Glc		*y*(±0.00669) = (0.01499 ± 0.00005)*x* + (−0.00647 ± 0.00251)	0.99986	0.067	0.223

^1^ Confidence level = 97%; ^2^ Regression coefficient, *r*^2^; ^3^ Limit of detection; ^4^ Limit of quantitation.

**Table 2:** Ginsenosides contents (mg/g) of *P. ginseng* grown at Bongwha, Korea in 2017.

Date	Total	Rg1	Re	Rf	Rg2	Rb1	Rc	Rb2	Rd	Ro
**21 January**	17.61 ± 0.17	2.22 ± 0.05	2.52 ± 0.06	0.64 ± 0.05	0.33 ± 0.05	5.28 ± 0.07	3.15 ± 0.05	1.72 ± 0.05	0.32 ± 0.06	1.44 ± 0.05
**4 February**	13.68 ± 0.19	1.25 ± 0.06	1.80 ± 0.07	0.38 ± 0.06	0.28 ± 0.06	3.93 ± 0.08	3.08 ± 0.06	1.70 ± 0.06	0.40 ± 0.07	0.85 ± 0.06
**24 February**	20.24 ± 0.24	1.80 ± 0.07	2.71 ± 0.08	0.59 ± 0.07	0.37 ± 0.08	6.12 ± 0.11	4.08 ± 0.08	2.78 ± 0.08	0.59 ± 0.09	1.20 ± 0.07
**2 March**	20.55 ± 0.24	1.75 ± 0.07	2.68 ± 0.08	0.62 ± 0.07	0.45 ± 0.07	6.22 ± 0.11	4.56 ± 0.08	2.38 ± 0.07	0.72 ± 0.09	1.17 ± 0.07
**9 March**	21.03 ± 0.25	1.70 ± 0.07	2.55 ± 0.08	0.60 ± 0.08	0.41 ± 0.08	6.69 ± 0.12	4.38 ± 0.08	2.87 ± 0.08	0.69 ± 0.09	1.14 ± 0.07
**24 March**	30.86 ± 0.23	1.64 ± 0.06	4.47 ± 0.08	0.68 ± 0.06	0.73 ± 0.06	9.69 ± 0.12	7.37 ± 0.08	3.80 ± 0.07	1.16 ± 0.07	1.30 ± 0.06
**7 April**	26.94 ± 0.26	2.43 ± 0.07	3.66 ± 0.09	0.79 ± 0.08	0.58 ± 0.08	7.99 ± 0.13	5.88 ± 0.09	3.10 ± 0.08	0.91 ± 0.07	1.60 ± 0.07
**19 April**	26.89 ± 0.26	2.46 ± 0.07	3.67 ± 0.09	0.74 ± 0.08	0.57 ± 0.08	7.94 ± 0.13	6.07 ± 0.09	2.75 ± 0.08	0.85 ± 0.09	1.85 ± 0.07
**9 May**	36.42 ± 0.26	3.30 ± 0.07	4.32 ± 0.08	1.00 ± 0.06	0.69 ± 0.06	11.22 ± 0.16	7.80 ± 0.10	4.29 ± 0.07	1.44 ± 0.07	2.37 ± 0.06
**19 May**	42.57 ± 0.26	3.69 ± 0.06	5.55 ± 0.08	1.30 ± 0.06	0.85 ± 0.06	13.51 ± 0.16	8.12 ± 0.09	5.31 ± 0.07	1.42 ± 0.07	2.83 ± 0.06
**10 June**	29.43 ± 0.27	2.94 ± 0.08	3.54 ± 0.09	0.87 ± 0.08	0.59 ± 0.08	8.93 ± 0.14	6.36 ± 0.09	3.30 ± 0.08	1.00 ± 0.09	1.91 ± 0.07
**23 June**	24.74 ± 0.25	2.89 ± 0.08	3.09 ± 0.09	0.77 ± 0.08	0.54 ± 0.08	7.31 ± 0.12	5.02 ± 0.08	2.72 ± 0.08	0.91 ± 0.09	1.49 ± 0.07
**6 July**	23.09 ± 0.25	2.66 ± 0.07	2.84 ± 0.08	0.64 ± 0.08	0.38 ± 0.08	6.45 ± 0.11	4.52 ± 0.08	3.22 ± 0.08	1.08 ± 0.09	1.29 ± 0.07
**22 July**	22.16 ± 0.25	2.64 ± 0.07	2.52 ± 0.08	0.71 ± 0.07	0.46 ± 0.08	6.77 ± 0.12	4.52 ± 0.08	2.27 ± 0.08	0.97 ± 0.09	1.30 ± 0.07
**5 August**	21.11 ± 0.25	2.70 ± 0.07	2.42 ± 0.08	0.65 ± 0.08	0.39 ± 0.08	6.07 ± 0.11	4.32 ± 0.08	2.51 ± 0.08	0.97 ± 0.09	1.08 ± 0.07
**17 August**	22.12 ± 0.25	2.55 ± 0.07	2.52 ± 0.08	0.76 ± 0.08	0.42 ± 0.08	6.20 ± 0.11	4.52 ± 0.08	2.87 ± 0.08	0.96 ± 0.09	1.32 ± 0.07
**1 September**	21.95 ± 0.20	2.39 ± 0.06	2.85 ± 0.07	0.62 ± 0.06	0.55 ± 0.06	6.37 ± 0.09	4.34 ± 0.06	2.76 ± 0.06	0.88 ± 0.07	1.19 ± 0.06
**14 September**	25.95 ± 0.26	2.85 ± 0.08	3.13 ± 0.09	0.72 ± 0.08	0.65 ± 0.08	7.49 ± 0.12	5.34 ± 0.09	3.39 ± 0.08	1.04 ± 0.09	1.34 ± 0.07
**2 October**	25.59 ± 0.26	2.66 ± 0.08	3.31 ± 0.09	0.71 ± 0.08	0.57 ± 0.08	7.27 ± 0.12	5.49 ± 0.08	3.06 ± 0.08	0.90 ± 0.09	1.63 ± 0.07
**14 October**	22.87 ± 0.25	2.91 ± 0.08	2.45 ± 0.08	0.85 ± 0.08	0.36 ± 0.08	6.75 ± 0.12	4.62 ± 0.08	2.76 ± 0.08	0.68 ± 0.09	1.47 ± 0.07
**26 October**	24.88 ± 0.25	2.53 ± 0.07	2.94 ± 0.09	0.74 ± 0.08	0.34 ± 0.08	6.86 ± 0.12	4.89 ± 0.08	3.90 ± 0.08	0.95 ± 0.09	1.72 ± 0.07
**10 November**	22.49 ± 0.25	2.16 ± 0.07	2.85 ± 0.08	0.74 ± 0.08	0.41 ± 0.08	6.36 ± 0.11	4.90 ± 0.08	3.01 ± 0.08	0.67 ± 0.09	1.40 ± 0.07
